# The Effect of Patient-related Factors Age, Sex, Implant Location, and Periodontitis on Crestal Bone Loss in the Posterior Ridge: A Retrospective Study 

**DOI:** 10.3290/j.ohpd.c_1869

**Published:** 2025-03-06

**Authors:** Yi Feng, Mengna Lin, Xiaofeng Wang, Fuming He

**Affiliations:** a Yi Feng Stomatology Hospital, School of Stomatology, Zhejiang University School of Medicine, Zhejiang Provincial Clinical Research Center for Oral Diseases, Key Laboratory of Oral Biomedical Research of Zhejiang Province, Cancer Center of Zhejiang University, Hangzhou, China. Conceived study idea, wrote the manuscript.; b Mengna Lin Stomatology Hospital, School of Stomatology, Zhejiang University School of Medicine, Zhejiang Provincial Clinical Research Center for Oral Diseases, Key Laboratory of Oral Biomedical Research of Zhejiang Province, Cancer Center of Zhejiang University, Hangzhou, China. Wrote the manuscript.; c; d Xiaofeng Wang Stomatology Hospital, School of Stomatology, Zhejiang University School of Medicine, Zhejiang Provincial Clinical Research Center for Oral Diseases, Key Laboratory of Oral Biomedical Research of Zhejiang Province, Cancer Center of Zhejiang University, Hangzhou, China. Collected and analysed the data.; e Fuming He Stomatology Hospital, School of Stomatology, Zhejiang University School of Medicine, Zhejiang Provincial Clinical Research Center for Oral Diseases, Key Laboratory of Oral Biomedical Research of Zhejiang Province, Cancer Center of Zhejiang University, Hangzhou, China. Conceived study idea.

**Keywords:** age factors, alveolar bone loss, dental implant, sex

## Abstract

**Purpose:**

To investigate the effects of patient-related factors such as age, sex, implant location, and history of periodontitis, on crestal bone loss in the posterior region throughout the surgical healing and functional periods.

**Materials and Methods:**

This study evaluated 311 implants from 163 patients, with an average follow-up of 27.10 months. Implants were assessed based on age, sex, implant location, and history of periodontitis. Crestal bone loss was quantified by measuring bone level changes using oral panoramic radiographs. Time T1 was defined as the period from implant placement to the healing phase, and T2 as the period from the second-stage surgery to the follow-up visit. Group comparisons were made using the Mann–Whitney U-test, with significance set at p < 0.05.

**Result:**

At T1, crestal bone loss averaged 0.27 ± 0.40 mm; at T2, it averaged 0.40 ± 0.50 mm. A statistically significant difference at T1 was observed between patients aged 20–39 and 40–59, and between these two age groups in female patients (p < 0.05). During T2, within the 40–59 age group, bone resorption differed statistically significantly between males and females (p < 0.05). Statistically significant differences were also noted between males aged 40–59 and those 60 years or older, and between females aged 20-39 and 40-59 (p < 0.05). There were no differences between the other groups.

**Conclusion:**

Crestal bone loss correlates with patient age and sex. Increased attention should be given to female patients within certain age ranges. Patients with history of periodontitis can maintain bone tissue stability around the implant.

The widespread use of dental implants has significantly improved the oral health-related quality of life.^
28
^ Peri-implant tissue stability is crucial for evaluating implant restoration success and has received widespread attention.^
18,47
^ Long-term implant success is ultimately gauged by limited, time-dependent alveolar bone loss, which should be clinically inactive and painless.^
50
^ Ensuring bone stability around the implant is essential for its mechanical security, functionality, and aesthetics. Crestal bone loss is a natural physiological change after implant placement.^
73
^ Systematic studies have indicated that alveolar bone loss for bone tissue level implants ranges from 0.5 mm to 0.8 mm after the implant begins to function.^
65
^ However, excessive bone resorption is considered a precursor to peri-implantitis.^
32
^ To mitigate bone resorption, researchers have explored various factors that may influence crestal bone loss. These factors include the materials and design of implants, the composition and structure of the restoration, surgical techniques, and overall systemic health.^
36
^ Nonetheless, the impact of many of these factors remains undetermined.^
5
^


Patient factors such as age, sex, history of periodontitis, smoking, and diabetes have been investigated for their roles in peri-implant bone loss.^
16,23,26
^ Despite attention to smoking and diabetes, research on age and sex remains scarce.^
1,2,7,9,10,30,57,75
^ Patients with poorly controlled diabetes suffer from impaired osseointegration, increased risk of peri-implantitis, and higher levels of implant failure.^
23,57
^ Smoking is harmful to tissues and plays a role in an individual’s immune and inflammatory response, wound healing, biofilm formation, and general health.^
10
^ A limited number of studies have noted differences in bone resorption and implant failure rates across age groups.^
6,12,26,43
^ A three-year clinical observation study focused on sex reported no statistically significant differences between sexes in crestal bone loss around posterior dental implants.^
58
^ However, other studies showed higher bone loss in females, especially after 24 months of loading.^
55
^ Additionally, research indicated peak bone resorption in women aged 50–60 years, attributing it to menopause.^
56
^ Presently, there is no consensus on how age and sex specifically impact crestal bone loss.

The incidence of peri-implant bone loss and peri-implantitis in the maxilla vs the mandible remains inconclusive.^
23,54
^ Some researchers have suggested no statistically significant difference exists in bone resorption between the mandibular and maxillary posterior regions.^
1,11
^ However, other studies have reported greater bone resorption in maxillary implants after a period of loading.^
30,56
^ The mandible, with higher bone density and less cancellous bone, is believed to bear loads more effectively and rebuild bone more slowly.^
56
^ Contradictory findings show that bone loss is most prevalent in the mandibular anterior region, followed by the mandibular posterior region, the maxillary anterior region, and least in the maxillary posterior region.^
6,71
^


The European Society of Periodontal Diseases has identified periodontal disease as a high risk factor for crestal bone loss.^
46
^ Clinical studies have supported this, showing that implants in patients with history of periodontitis tend to experience more bone loss, increasing the risk of peri-implantitis and implant failure.^
21,67
^ This is attributed to a shared bacterial aetiology between peri-implantitis and periodontitis, with similar anaerobic bacteria found around both periodontally affected teeth and implants with bone loss.^
20,78
^ Conversely, some researchers believe that crestal bone loss is not related to the progression of periodontitis.^
19
^ Considering the disparities between the implant interface and natural periodontal tissues, the mechanism of peri-implant asymptotic marginal bone resorption differs from that of natural teeth.^
78
^ Therefore, a definitive link between bone resorption in natural teeth and implants has yet to be identified.^
77,78
^


Researchers generally agree that bone loss should not occur during the submerged healing phase.^
3,49
^ While this type of bone resorption does not immediately threaten implant success, it may jeopardize long-term stability.^
39,68
^ However, bone resorption has been observed in clinical studies, with an average loss of 0.2 to 0.5 mm reported before second-stage surgery in implants without bone grafting.^
13,44
^ The causes of initial bone loss in submerged implants are not entirely clear but are frequently attributed to surgical trauma and various patient risk factors.^
68
^ After surgery, the inflammatory response leads to bone demineralisation, which may result in crestal bone loss during the healing process.^
41
^ Researchers examining the role of patient characteristics on bone resorption are currently looking at molecular factors, such as interleukin-1.^
3
^ As for broader patient-related factors—age, sex, implant location, and history of periodontal disease—studies on their influence on early implant bone loss are scarce.^
13,49,69
^


The present research hypothesis is that patient-related factors such as gender, age, implant position, and history of periodontal disease are affected by crestal bone loss. By identifying these factors, this study seeks to provide more personalised patient management and improve strategies for oral hygiene education.

## Materials and Methods

This retrospective study was approved by the Ethics Committee of Zhejiang University, Hangzhou, China (No: 2022103).

In this study, patients who visited the Stomatology Hospital (School of Stomatology, Zhejiang University School of Medicine) from 2012 to 2016 and underwent implantation with Straumann bone-level implants (Straumann; Basel, Switzerland) and implant-supported prosthesis were selected to voluntarily participate. Their implant surgeries were performed by the same experienced surgeon. After administering local anesthesia, a full-thickness flap was turned over to fully expose the surgical area, and Straumann bone-level implants (3.3-4.8 mm in diameter and 8–14 mm in length) were placed. Three months post-surgery, after confirming osseointegration, the upper restoration was carried out, selecting a suitable finished abutment. The crown material was chosen based on clinical requirements and patient preference.

Eligible participants met the following inclusion criteria:^
72
^ (1) good general health; (2) sufficient bone height and width for implantation; (3) voluntary participation in this study. The exclusion criteria encompassed: (1) systemic health issues that contraindicate the procedure (e.g., uncontrolled endocrine disorders, metabolic bone diseases, or a history of severe treatments such as radiotherapy or chemotherapy); (2) suboptimal oral hygiene; (3) ongoing periodontal infection; (4) the presence of bruxism; (5) a heavy smoking habit defined as smoking more than 10 cigarettes daily.

We meticulously gathered pertinent demographic information on all patients, encompassing sex, age at the time of dental implantation, history of periodontal disease, smoking status, and presence of systemic diseases such as diabetes, cardiovascular disease, etc. Panoramic radiographs were obtained pre-operatively, post-operatively, before the secondary stage surgery, and during the post-restoration follow-up period. The plaque index was recorded and the average probing depth was measured using a Williams probe at six surfaces of the implant: mesiobuccal, midbuccal, distobuccal, mesiolingual, midlingual, and distolingual. Peri-implant infection was evaluated by checking for bleeding-on-probing during the first 30 s (0 = absent, 1 = present). The medical records were examined for any special intraoral conditions during follow-up, such as redness and swelling of the gingiva around the implant, discharge of pus, etc. The changes in alveolar bone height over time were carefully measured by the same dentist.

**Fig 1 fig1:**
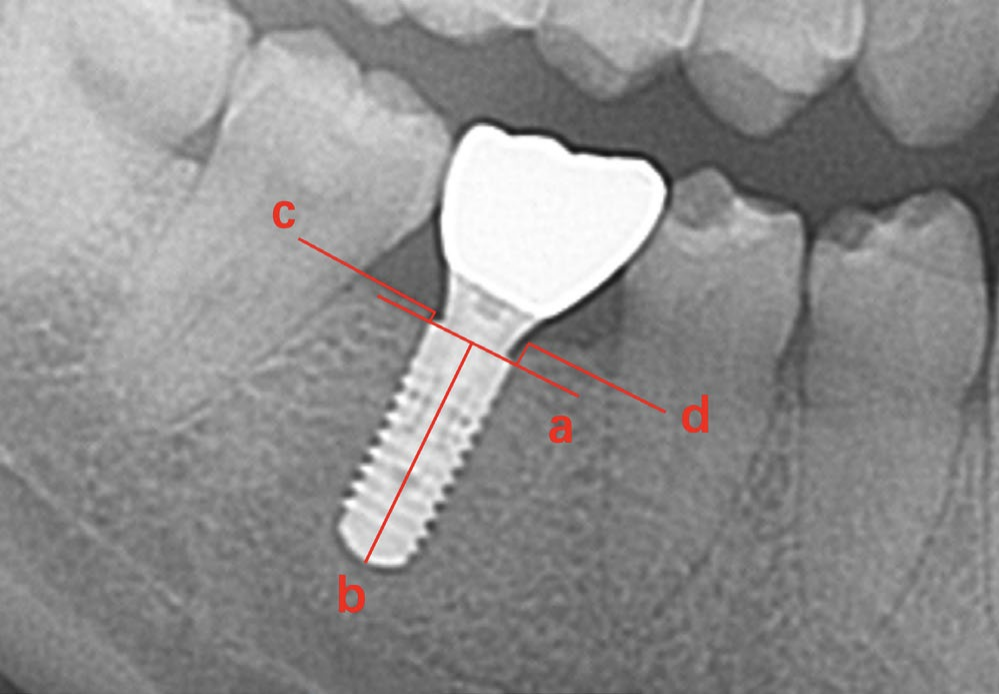
(a) Line through the implant platform. (b) A perpendicular line aligned with the implant’s long axis, orthogonal to line (a). (c) The line in the distal direction of the implant, passing through the most coronal point of the bone at the edge of the implant and parallel to line (a). (d) Located in the mesial direction of the implant, passing through the most coronal point of the bone at the edge of the implant and parallel to line (a).

Oral panoramic radiography (Orthopantomograph OP 200 D; Instrumentarium Imaging; Tuusula, Finland) was used to assess bone resorption of the peri-implant tissue. Reading of the panoramic radiographs was performed by Clinview Software (Clinview Software, 6.1.3.7 Version; Instrumentarium Imaging). The measurement method is shown in Fig 1.

In the recorded images, the distances between line “c” and line “a”, as well as line “d” and line “a” were the distal and mesial marginal bone levels of the implant, respectively (Fig 1). The distance was corrected by the known implant length.

The degree of bone resorption during the postoperative healing phase (T1) was quantified by comparing panoramic radiographic images taken before the second-stage surgery with those captured immediately following the implant procedure. Similarly, the extent of bone resorption during the loading phase (T2) was determined by the discrepancy between the panoramic images obtained prior to the secondary surgery and those taken during the follow-up visit.

Statistical analysis was conducted at both the patient and implant levels using SPSS software (SPSS R26.0.0.0; Chicago, IL, USA). The Kolmogorov-Smirnov test was used to assess data normality. Descriptive statistical results were expressed as mean, standard deviation, median, and interquartile ranges. The Mann-Whitney U-test was used as a non-parametric test for comparison between groups. The Kruskal-Wallis test was used to compare three or more time groups, for non-normally distributed data. Statistical significantce was set at a p-value of 0.05 in all tests.

## Results

A total of 163 patients, aged from 23 to 85 years, with an average age of 50.0, participated in the study. Among them were 71 males and 92 females, with 311 implants included. The average follow-up period since the second stage was 27.1 months. Of these patients, 79 presented with a history of periodontitis. Eight patients with 18 implants had a history of hyperglycemia, and six patients with 11 implants had a history of smoking. 235 implants were placed in the posterior mandibular region, and 76 were placed in the posterior maxillary area.

**Fig 2 fig2:**
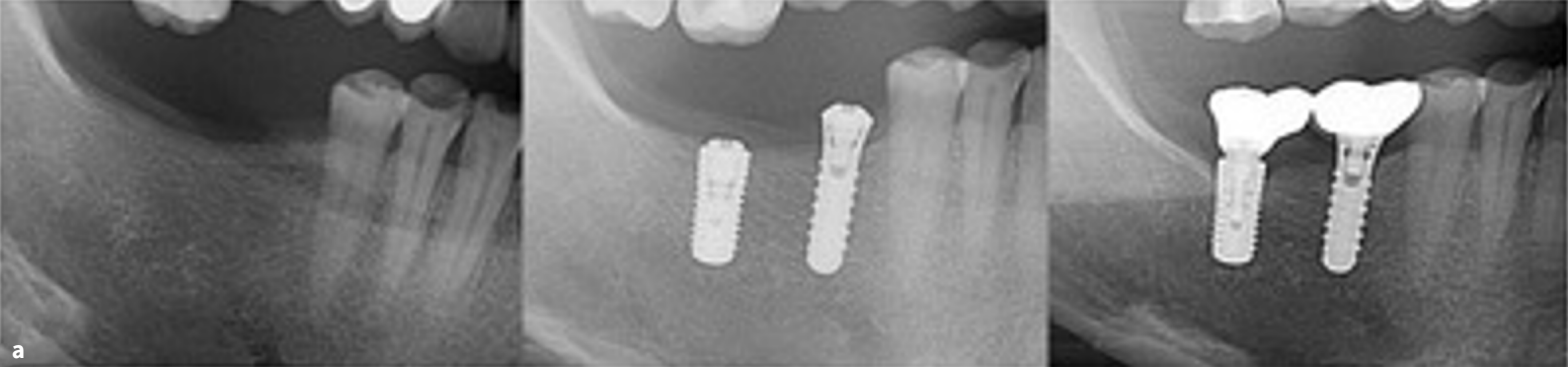

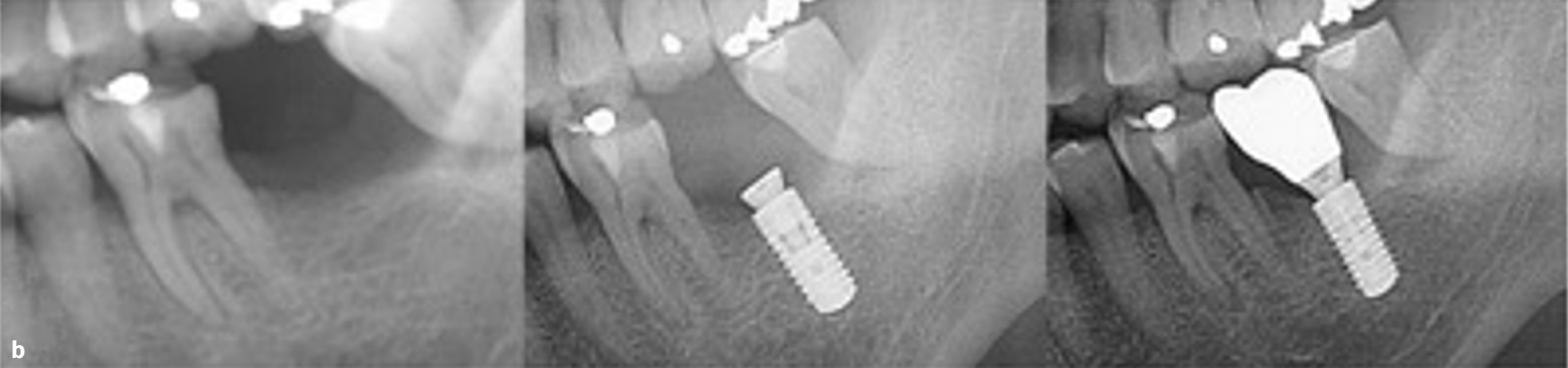
Radiographs taken (a) 40 months after loading, showing statistically significant bone resorption around the implant and (b) 33 months after loading, when the peri-implant bone level was stable.

During the healing phase (T1), average bone resorption was 0.27 ± 0.40 mm, with a 50^th^ quartile of 0.20 mm (25^th^ quartile 0.05, 75^th^ quartile 0.40). In the loading phase (T2), average bone resorption was 0.40 ± 0.50 mm and the 50^th^ quartile was 0.30 mm (25^th^ quartile 0.05, 75^th^ quartile 0.65). An analysis from T2 revealed that 64.6% of implants showed bone resorption of 0.5 mm or less, and 91.6% had no more than 1 mm. Only 4 implants exhibited statistically significant resorption > 2 mm during follow-up. Typical images of bone resorption and stable bone levels around the implant during the follow-up are shown in Fig 2.

**Fig 3 fig3:**
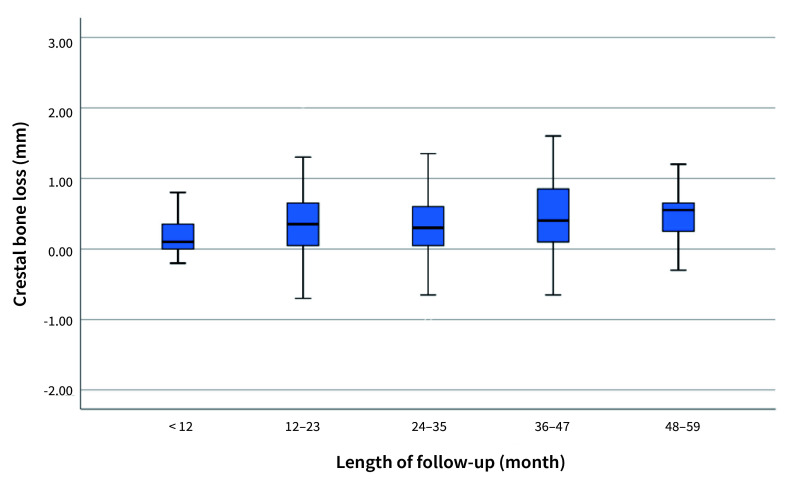
Crestal bone loss for implants with different lengths of follow-up (T2).

The implants were categorised by follow-up interval, and corresponding bone resorption box plots were created, as shown in Fig 3. No statistical differences were observed between the different time groups.

In this study, we placed 125 implants in male patients and 186 in female patients. During T1, the mean alveolar bone resorption was 0.29 ± 0.45 mm in males and 0.26 ± 0.35 mm for females, a difference that was not statistically significant (p = 0.911). After loading, average bone resorption was 0.38 ± 0.55 mm for males and 0.41 ± 0.46 mm for females, with no statistically significant difference (p = 0.214). Implants were distributed among three age groups: 20–39 years (89 implants), 40–59 years (136 implants), and over 60 years (86 implants). Stastically significant bone resorption differences were observed at T1 between the 20–39 and the 40–59 age groups (p = 0.000), but not between 40–59 and the ≥ 60 age groups. The comparison of age groups during the T2 observation period revealed no statistically significant differences. Implant location included 76 implants in the maxilla and 235 in the mandible, with no statistically significant difference in bone resorption between these groups (p > 0.05). Patient periodontal history was also assessed, revealing 153 implants in healthy periodontal patients and 158 in those with a history of periodontitis, which was not statistically significantly different (p > 0.05). Detailed values are presented in Table 1.

**Table 1 table1:** Crestal bone loss (CBL) in patients of different sex, age, different implant positions, and periodontal history

	Number	CBL(T1)	p-value	CBL(T2)	p-value
Mean ± SD	Median (25^th^ percentile, 75^th^ percentile)	Mean ± SD	Median (25^th^ percentile, 75^th^ percentile)
**Sex**							
	Male	125	0.29 ± 0.45	0.20 (0.05, 0.40)	0.911	0.38 ± 0.55	0.30 (0.00, 0.63)	0.214
	Female	186	0.26 ± 0.35	0.20 (0.05, 0.40)		0.41 ± 0.46	0.35 (0.10, 0.70)	
**Age**							
	20-39	89	0.18 ± 0.38	0.15 (0.00, 0.25)	0.000^#*^	0.41 ± 0.56	0.25 (0.05, 0.65)	0.949#
	40-59	136	0.33 ± 0.37	0.25 (0.10, 0.45)	0.155^##^	0.36 ± 0.46	0.30 (0.05, 0.60)	0.186##
	≥ 60	86	0.29 ± 0.44	0.18 (0.05, 0.40)		0.45 ± 0.50	0.40 (0.10, 0.75)	
**Position**							
	Maxilla	76	0.21 ± 0.34	0.15 (0.00, 0.48)	0.132	0.46 ± 0.53	0.30 (0.10, 0.75)	0.542
	Mandible	235	0.29 ± 0.41	0.20 (0.05, 0.40)		0.38 ± 0.49	0.35 (0.05, 0.65)	
**History of periodontitis**
	Periodontally healthy patients	153	0.29 ± 0.35	0.25 (0.08, 0.40)	0.189	0.42 ± 0.46	0.35 (0.10, 0.65)	0.496
	Treated periodontitis	158	0.26 ± 0.44	0.15 (0.04, 0.40)		0.38 ± 0.53	0.30 (0.05, 0.66)	
T1: from implant surgery to the time of the second stage surgery; T2: from the time of the second stage surgery to the return visit. *Mann-Whitney U-test, p < 0.05. ^#^, p-value*(20-39×40-59); ^##^, p-value*(40–59×≥ 60).

During T1, a statistically significant difference in bone resorption was observed between female patient groups aged 20-39 and those aged 40–59 (p = 0.001). However, no statistically significant differences were observed in other age groups (p > 0.05). Specific values are detailed in Table 2.

**Table 2 table2:** Statistical table of the effect on CBL (T1) of different sex and ages

Age	Male	Female	p-value* (male× female)
Number	Mean ± SD	Median (25^th^ percentile, 75^th^ percentile)	Number	Mean ± SD	Median (25^th^ percentile, 75^th^ percentile)
20–39	32	0.20 ± 0.47	0.20 (0.04, 0.34)	57	0.17 ± 0.32	0.10 (0.00, 0.25)	0.625
40–59	60	0.34 ± 0.40	0.20 (0.10, 0.44)	76	0.32 ± 0.35	0.33 (0.10, 0.50)	0.951
≥ 60	33	0.28 ± 0.53	0.15 (0.00, 0.30)	53	0.29 ± 0.37	0.25 (0.08, 0.55)	0.267
p-value* (20–39× 40–59)			0.104			0.001*	
p-value* (40–59× ≥ 60)			0.103			0.714	
*Mann-Whitney U-test, p < 0.05.

After loading, no statistically significant difference in bone resorption was noted between male and females within the 20–39 years age group (p > 0.05). However, in the 40–59 age group, bone resorption was 0.26 ± 0.45 mm for males and 0.44 ± 0.44 mm for females, indicating a difference between the groups (p = 0.004). Among patients aged 60 years or older, the difference in implant-neck bone resorption between males and females was not statistically significant (p > 0.05). No difference was noted in bone resorption between the 20- to 39- and 40-to 59-year age groups among male patients (p > 0.05), but there was a statistically significant difference between the 40–59 and ≥ 60 age group (p = 0.046). In female patients, a statistically significant difference was observed in bone resorption between the 20–39 and 40–59 age groups(p < 0.05), but no statistically significant difference was noted between the 40–59 and ≥ 60 groups (p > 0.05). The details are shown in Table 3.

**Table 3 table3:** Statistical table of the effect on CBL (T2) of different sex and ages

Age	Male	Female	p-value* (male× female)
Number	Mean ± SD	Median (25^th^ percentile, 75^th^ percentile)	Number	Mean ± SD	Median (25^th^ percentile, 75^th^ percentile)
20–39	32	0.58 ± 0.77	0.38 (0.01, 0.95)	57	0.32 ± 0.37	0.25 (0.05, 0.58)	0.192
40–59	60	0.26 ± 0.45	0.23 (0.00, 0.50)	76	0.44 ± 0.44	0.45 (0.11, 0.70)	0.004*
≥ 60	33	0.41 ± 0.40	0.40 (0.05, 0.75)	53	0.47 ± 0.56	0.40 (0.10, 0.73)	0.915
p-value* (20–39× 40-59)			0.051			0.041*	
p-value* (40–59× ≥ 60)			0.046*			0.814	
*Mann-Whitney U-test, p < 0.05.

## Discussion

This retrospective study focused on the changes in peri-implant bone tissue under vaious patient conditions 1-5 years after restoration. The findings indicated that individuals with effectively managed periodontal disease tended to maintain favourable peri-implant hard tissue conditions in the short to intermediate term. Additionally, the study noted that menopausal women were more prone to increased bone resorption during both the surgical healing and loading phases.

In this investigation, mean bone resorption during the healing phase was 0.27 mm, and 0.25 mm during the loading phase, closely aligning with other clinical studies. The literature suggests that the typical range of bone resorption during the submerged healing phase lay between 0.18 mm and 0.5 mm.^
13,44
^ After loading, a three-year study on Straumann bone-level implants documented bone resorption rates of 0.30 mm to 0.45 mm.^
4
^


The findings of this study showed no statistically significant differences in bone resorption between female and male patients, consistent with existing studies.^
13
^ During the healing phase, women within a specific age range exhibited increased bone resorption around the implant; the same age group showed more bone resorption compared to men during the loading phase. This age group coincided with menopause, a critical period that typically begins in women’s 40s, with the median age of 51 for the complete cessation of menstruation.^
31
^ Estrogen deficiency increases osteoclast activity and decreases osteoblast activity.^
8,31
^ Animal studies showed a decrease in the volume of cancellous bone around implants and the bone-implant contact area, especially in scenarios where estrogen levels statistically significantly drop after implant osseointegration or in subjects with inherently low estrogen levels.^
31,34,59
^ Researchers have postulated that aging women might experience increased crestal bone loss due to hormonal fluctuations.^
55
^ Concurrently, the oral environment also undergoes considerable changes, including decreased saliva flow and pH level in menopausal women.^
29
^ A five-year prospective study on periodontitis and alveolar bone resorption in postmenopausal women revealed variations in subgingival microbiota correlated with the progression of periodontal disease.^
45
^ Despite these findings, clinical research has not established a direct link between menopause and the occurrence of peri-implantitis.^
22
^ Based on this evidence, personalised patient education and treatment plans are recommended for menopausal patients, with more frequent follow-ups to closely monitor alveolar bone loss.^
69
^ For patients who are already experiencing symptoms of menopause, such as dry mouth, frequent sips of water may be recommended, or the use of small sugar-free candies to increase saliva production.^
29
^


In this study, we observed no statistically significant difference in peri-implant bone loss between younger and older patients, which agrees with Bryant and Hoeksema’s research.^
16,38
^ It is a common belief that aging generally means a compromise of the healing potential of soft tissues and bones.^
14,17
^ Age-related bone loss predominantly affects cancellous bone, and the increase in oxidative stress associated with aging primarily stimulates osteoclastic activity on the trabeculae.^
51
^ At the same time, periodontal and peri-implant diseases are more common in older individuals.^
23
^ Age-related causes of increased periodontal infections may be linked to compromised oral hygiene due to reduced dexterity and vision loss.^
23
^ Despite less plaque accumulation on implants, the peri-implant mucosa shows a more pronounced clinical response compared to gingiva around natural teeth.^
52
^ Therefore, implant restorations for the elderly should emphasise ease of maintenance to promote oral hygiene and ensure implant stability.^
66
^


The study also observed no statistically significant difference in crestal bone loss between the maxilla and mandible, which concurs with some clinical research.^
1,11,58
^ However, previous studies indicated that maxillary and mandibular bones differ in their remodeling ability and rate.^
60
^ The maxillary region is characterised by robust vascularisation and strong reconstruction potential after implant placement, while the mandibular response tends to be more gradual.^
58
^ Additionally, the bone absorption ratio is positively correlated with the presence of cortical bone.^
37
^ This is also the reason why some authors have found more bone resorption and a higher incidence of peri-implantitis in the anterior region.^
54
^ Thin cortical bone prevents dissipation of bite force and increases stress around the implant.^
54
^ Trabecular bone is vital in distributing masticatory forces and reducing microfracture risks.^
24,40
^ Concurrently, cancellous bone, with its superior vascularisation and tissue repair abilities, contributes much to the healing process.^
24,40
^ Consequently, some researchers recommended a meticulous evaluation of the cortical:cancellous bone ratio, especially when implanting in the mandible’s posterior regions, to anticipate potential early bone resorption surrounding the implant.^
70
^


This study found that patients with a history of periodontal disease did not experience greater bone resorption compared to healthy individuals, contradicting many published findings.^
33,74
^ However, some studies pointed out that because of the persistent and cumulative effect of history of periodontitis factors on bone resorption, the difference would not be apparent until 50 months later,^
48
^ which could explain our results. Additionally, the severity of periodontal disease history, particularly a history of widespread aggressive periodontitis, influences the extent of bone resorption.^
25
^ Patients with a history of extensive aggressive periodontitis show statistically significantly more peri-implant bone resorption than those with a history of chronic periodontitis.^
74
^ Notably, our sample did not include participants with a history of generalised aggressive periodontitis. Moreover, the patients in this study requiring periodontal treatment had to undergo it before implant surgery; implant restoration was conducted only after their periodontal health stabilised. This rigorous treatment regimen and continuous oral hygiene education presumably enhanced oral health awareness, contributing to a positive prognosis for both natural teeth and implants.^
15,35,79,80
^ A large-scale retrospective study of 4951 implants found no significant influence of periodontal history on crestal bone loss when periodontal conditions were meticulously treated and monitored over time.^
30
^ Studies with up to 20 years of observation have confirmed that supportive periodontal care for implant patients with a history of periodontitis helps achieve high long-term survival and reduces the risk of peri-implant diseases.^
27,62,63
^ Personalised periodontal-care guidance can even be provided through online tools.^
61
^


Finally, many have postulated that bone resorption at the implant neck may primarily be an immune response to the implant itself, differing fundamentally from the resorption mechanism associated with periodontitis.^
5,76,78
^ This distinction underscores the complex nature of peri-implant bone changes and highlights the need for further research in this area.

The main limitations of this study were the reliance on two-dimensional panoramic data, which did not adequately capture changes in bone tissue on the labial and buccal sides of the implant. Using cone-beam computed tomography (CBCT) could degrade image quality due to artifacts from implants and other high-density materials, complicating bone measurement.^
42,64
^ Second, the use CBCT imaging as a review method exposes patients to a higher level of radiation.^
42
^ The use of CBCT as a diagnosis of bone resorption must be very clearly indicated and justified in terms of radiation dose and economic considerations.^
53
^ Based on these considerations, we chose two-dimensional images as the measurement method for this study.

## Conclusion

Within the limitations of this study, a combined effect of sex and age on bone resorption was found. Female patients in a certain age group, i.e., around menopause, require more attention. Patients with a history of periodontitis could also maintain peri-implant bone stability with proper treatment and good oral hygiene.

## References
